# Rotation and transportation of liquid crystal droplets for visualizing electric properties of microstructured electrodes

**DOI:** 10.1038/s41598-023-31026-8

**Published:** 2023-03-16

**Authors:** Shinji Bono, Satoshi Konishi

**Affiliations:** 1 Department of Mechanical Engineering, College of Science and Engineering, Ritsumeikan University, Kusatsu, 525-8577 Japan; 2 Graduate Course of Science and Engineering, Ritsumeikan University, Kusatsu, 525-8577 Japan; 3 Ritsumeikan Advanced Research Academy, Kyoto, 604-8502 Japan; 4 Ritsumeikan Global Innovation Research Organization, Ritsumeikan University, Kusatsu, 525-8577 Japan

**Keywords:** Electrical and electronic engineering, Liquid crystals

## Abstract

The spatial resolution of typical sensor probes is sufficient for measuring the average electric properties of microelectrical devices, but they are unable to measure the distribution with a spatial precision. Liquid crystal droplets (LCDs) are promising candidate for visualizing the distribution. When voltage is applied, the LCDs show rotational and translational behaviors which depend on the location of LCDs within the devices. We demonstrate that by comparing the experimental and numerical results, the electric field and electrostatic energy distribution are visualized by rotating and transporting LCDs, with a spatial resolution of 10 µm and a detection accuracy of 5 µV/µm. In addition, we produced an array of LCDs by designing periodic modulation of the electrostatic energy density in the model device. These findings show that the LCDs serve as a periodic modulator of the refractive index as well as a sensor for the observation of electric properties of microelectrical devices.

## Introduction

In microelectromechanical systems (MEMS), sensors and actuators are the core and essential components^1,2^ and recently have enabled the integration of micrometer-sized sensors and actuators on chips^3,4^. The development of integrated microelectrical tools capable of simultaneously performing analysis and regulation has been achieved. However, prior to the fabrication of the integrated microelectrical devices, the physical properties of the devices are simulated using numerical analysis, and the design of the devices is optimized to ensure proper operation^5,6^. In addition, defects and degradation during the manufacturing processes and regular and continuous use result in loss of functionality and critical accidents. To improve the yield and safety, it is critical to identify the source of these errors to rectify it; however, it is challenging to incorporate boundary faults conditions in numerical calculation because the structure of produced devices with faults differs from that of the design. To retain a micrometer-scaled spatial resolution, sensing the physical quantity, such as electric field, in fabricated microelectrical devices has received significant attention^6^.

Previously, physical parameters such as the electrostatic potential^7^ have been measured using sensor probes bigger than the typical length scale of microdevices. The spatial resolution of these probes is in the order of micrometer, which is sufficient for measuring the average value of physical quantities across the entire device, but they are unable to measure the distribution of such values with a spatial precision. Thus, we can detect the presence errors, but not the location in manufactured microelectrical devices. A sensing detector with a high detection accuracy spatial resolution is critical to feedback the location of faults to device design. To visualize physical values in a microdevice with great spatial resolution, significant efforts have been concentrated on liquid crystal droplets (LCDs) as sensors^8–11^. Soft materials with molecular orientational order are called liquid crystals (LCs) and are known to respond to external stimuli, including heat, electric field, and magnetic field^12^. LCDs spontaneously disperse when the LC samples on microelectric devices are cooled from the isotropic (Iso) phase temperature to the LC-Iso coexisting phase temperature and disperse spontaneously^13,14^. LCDs offer great spatial resolution that is comparable to the typical size of LCDs (~ 10 µm) as well as a good detection accuracy that reflects the LCs’ strong responsiveness to external stimuli. Previous studies have proposed heat flow sensors based on the cross-correlation of cholesteric (Ch) LCs with broken mirror symmetry; LCDs on microelectrical devices may detect microtemperature gradient (~ 0.1 mK/µm) with a high spatial resolution (~ 10 µm)^8,9^.

In addition to heat flow, LCDs respond to electric field, when applied to bulk LCs with positive dielectric anisotropy, the electric field torque to spin the molecule orientation parallel to the line of electric force. Thus, for LCDs in the presence of electric field, the torque required to spin the main axis parallel to the direction of the electric field must be applied^15^. In addition, because the LCs exhibit birefringence, polarized optical microscopy (POM) can be used to determine the orientation of the primary axis^12,16^. As a result, LCD is a promising candidate for particle imaging electrometry (PIE), which involves the visualization of the electric field of a microelectric device with a high spatial resolution (~ 10 µm), and the information measured by LCDs can be used to determine the source of defects in microelectronic devices. LCD-based PIE must improve the performances of electrostatic actuators^2,6^, digital microfluidics^17^, and electrophoresis in lab-on-a-chip^18^. In this article, we propose an LCD-based PIE mechanism for micrometer-scaled spatial resolution visualization of the electric field distribution on microstructured electrodes. Initially, LCDs with dielectric anisotropy were dispersed on microcomb electrodes considered as an electrostatic actuator with applied voltage. This was followed by evaluation of the rotation and transport of LCDs to visualize the electric field and electrostatic energy distribution. Furthermore, electrostatic energy distribution in the microcapacitive device was periodically modulated. The translational motion of LCDs was regulated by adjusting the electrostatic force and produced by the micrometer-scaled array structure of LCDs, which can be regarded as a refractive index modulation mechanism as well as a visualization mechanism of electric properties in microstructured electrodes.

## Results

### Visualization of direction and magnitude of electric field in microcomb electrodes

**Figure 1:**
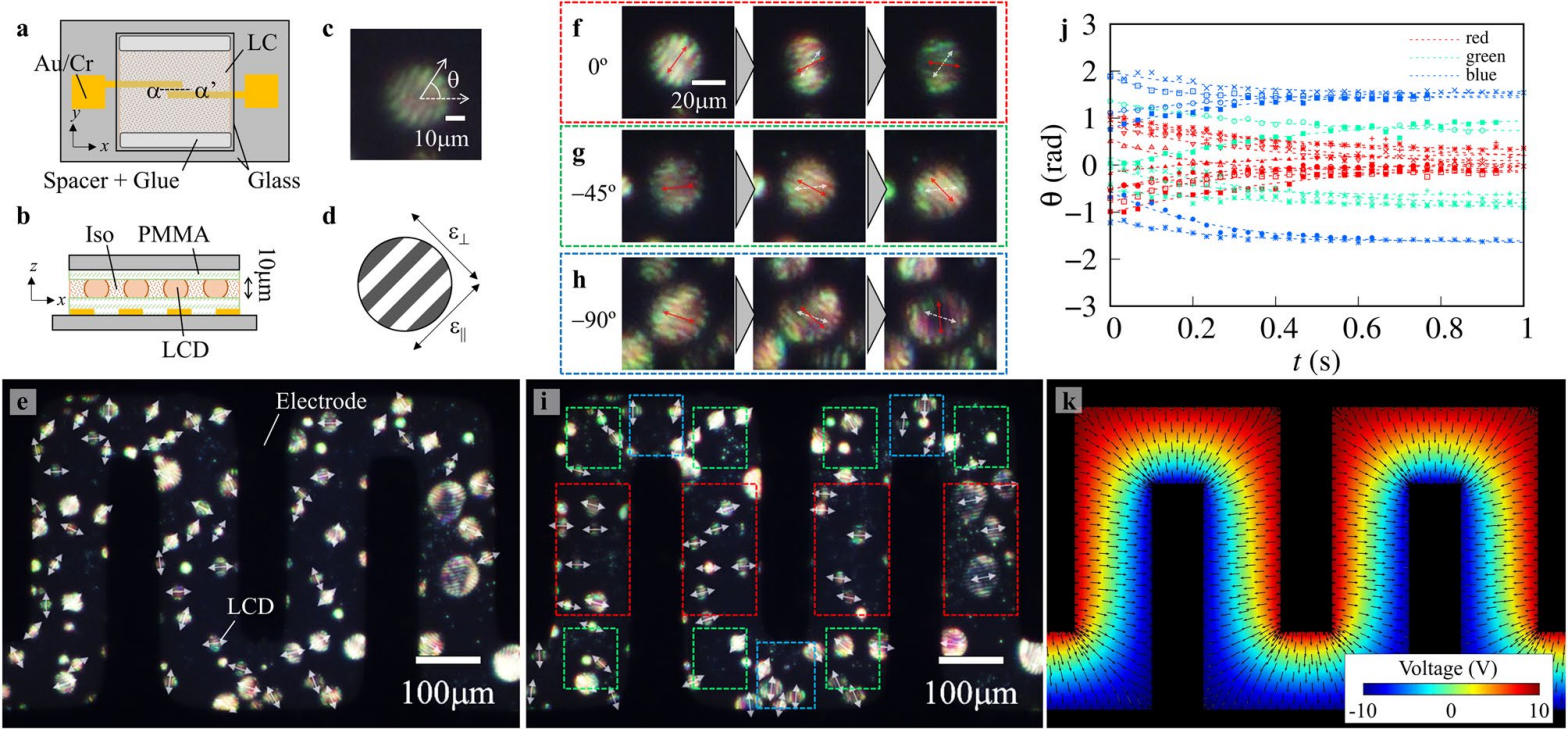
The rotational behavior of LCDs on microcomb electrodes. **(a)** Top view schematic illustration of the model device. We defined *x*- and *y*-axes as shown in this figure. **(b)** Cross-sectional view (α–α′) of the model device. We patterned comb-Au/Cr electrodes on the lower glass substrate and dispersed LCDs just above the electrodes. The thickness of the cell was 10 µm. **(c)** POM images of an LCD. We defined the angle between the *x*-axis and the striped texture as θ. **(d)** Schematic illustration showing the dielectric anisotropy of LCD. Dielectric constants parallel and perpendicular to the striped texture and denoted as ε_||_ and ε_⟂_, respectively. The LC sample has positive dielectric anisotropy (Δε = ε_||_− ε_⟂_ > 0). **(e)** POM images of LCD on microcomb electrodes before voltage application, and white arrows indicate the direction of ***n***. POM images of LCDs after voltage application with θ_∞_ = **(f)** 0°, **(g)** − 45°, and **(h)** − 90°. Snapshots were taken every 2/3 s. We represent ***n***(*t*) and initial ***n***(*t* = 0) as red arrows and white dashed arrows, respectively. **(i)** POM image of LCDs after voltage (20 V, 10 kHz) application for 5 s, and white arrows indicate ***n***. We divide the observation area into three zones surrounded by red, green, and blue lines depending on the equilibrium direction of LCDs. **(j)** Time evolution of θ. Red, green, and blue markers indicate the θ(*t*) of LCD at the corresponding area of **(i)**. **(k)** Voltage distribution on the microcomb electrodes calculated numerically by COMSOL. The color map indicates the electric potential. We showed the direction of electric fields as arrows.

The electric field distribution on the microcomb electrodes, which were used as the electrostatic capacitive actuators, was first evaluated. Figure 1a and 1b display the device’s top view schematic illustration and α–α′ cross-section, respectively. The LCDs were dispersed above the electrode detected by the electric field generated from microcomb electrodes patterned on the lower glass substrate. The LCDs are randomly dispersed on the microcomb electrodes when the LC cell is cooled from the Iso temperature phase to the LC-Iso coexisting temperature phase. As shown in Fig. 1c, the LCDs have a striped texture with a spacing of ~ 10 µm. A Ch-LC mixture of host nematic LC and 0.93 wt.% of chiral dopant was used. A single-twist (ST) helix is known to be the director configuration in 10-µm-sized LCDs. The helical axis is perpendicular to the striped texture, and the spacing corresponds to half pitch of the helix^19,20^. The angle between the stripes and the *x*-axis is θ as shown in Fig. 1a. Due to the positive dielectric anisotropy of the host LCs, the dielectric anisotropy of LCDs is positive as shown in Fig. 1d. Δε = ε_||_ − ε_⟂_, where ε_||_ and ε_⟂_ are the parallel and perpendicular dielectric constants of the striped texture, respectively. The direction of the principal axis of Δε is defined as a pseudo vector ***n***, where ***n*** has head-to-tail symmetry (***n*** =  − ***n***).

A micrograph of the LCDs dispersion on the microcomb electrodes using POM is shown in Fig. 1e. Although, the Au/Cr electrodes do not have the ability to transmit illuminated light, they are represented by the gray and bright regions corresponding to the Iso phase and optically anisotropic LCDs, respectively^8,9^. The directions of θ are indicated with white arrows, and in the absence of an electric field, the distribution θ is completely random. When an alternative current (AC) of 20 V and a frequency of 10 kHz are applied to microcomb electrodes, rotating dispersing LCDs are produced (Supplementary Movie 1). Figure 1f displays the rotational behavior of an LCD between a pair of electrodes that are parallel to the *y*-axis. The direction θ(*t*) and the initial direction θ_0_ are represented with red and white two-way arrows, respectively, and the LCD rotates counterclockwise (CCW) when a voltage is applied and reached equilibrium direction θ_∞_ ~ 0° after 2 s. The electrostatic energy density of an ST-LCD is given as:

1 $${\text{F}}_{{{\text{el}}}} = - \frac{1}{2}\upvarepsilon _{0}\upvarepsilon _{ \bot } E^{2} - \frac{1}{2}\upvarepsilon _{0} \Delta\upvarepsilon \left( {{\varvec{n}} \cdot {\varvec{E}}} \right)^{2}$$

where ***E*** and ε_0_ are the electric field and the dielectric constant of the vacuum, respectively^12^. When ***n*** and ***E*** are parallel, *F*_el_ is minimized and directly corresponds to the direction of the line of electric force.

LCDs POM images are shown in Fig. 1g and 1h and are enclosed by green and blue dashed lines, respectively, in Fig. 1i. The POM images of LCDs on microcomb electrodes in Fig. 1i were obtained after 5 s of voltage exposure. The observation area was divided into three regions; the equilibrium directions of LCDs θ_∞_ in red, green, and blue regions are 0°, ± 45°, and ± 90°, respectively. The time evolution of θ is shown in Fig. 1j, indicating that θ reaches θ_0_ within 1 s and θ_∞_ is not dependent on θ_0_ but rather on the location of LCDs on the microcomb electrodes. The rotational direction is determined by θ_0_, and in the presence of an applied voltage, the LCDs rotate to minimize the deviation as θ_∞_ decreases. Thus, LCDs with tan θ_0_ > 0 (< 0) have a clockwise (CW) rotation, and the equilibrium direction of LCDs surrounded by a green (blue) line is ± 45° (± 90°) due to the head-to-tail symmetry.

A commercial multiphysics software (COMSOL Multiphysics®) was used to conduct numerical analysis of the electric field on microcomb electrodes. In Fig. 1k, a color-coded distribution of electrical potential on the microcomb electrodes is presented, and the line of an electric field is estimated as arrows and superimposed using the electrical potential distribution. The directions of an electric field in red, green, and blue rectangles defined in Fig. 1i are 0°, ± 45°, and ± 90°, respectively, which is consistent with the experimental results. The agreement between the experimental and numerical calculation demonstrates that the electric field distribution can be visualized by evaluating the orientation of LCDs placed on a microelectrical device.

**Figure 2:**
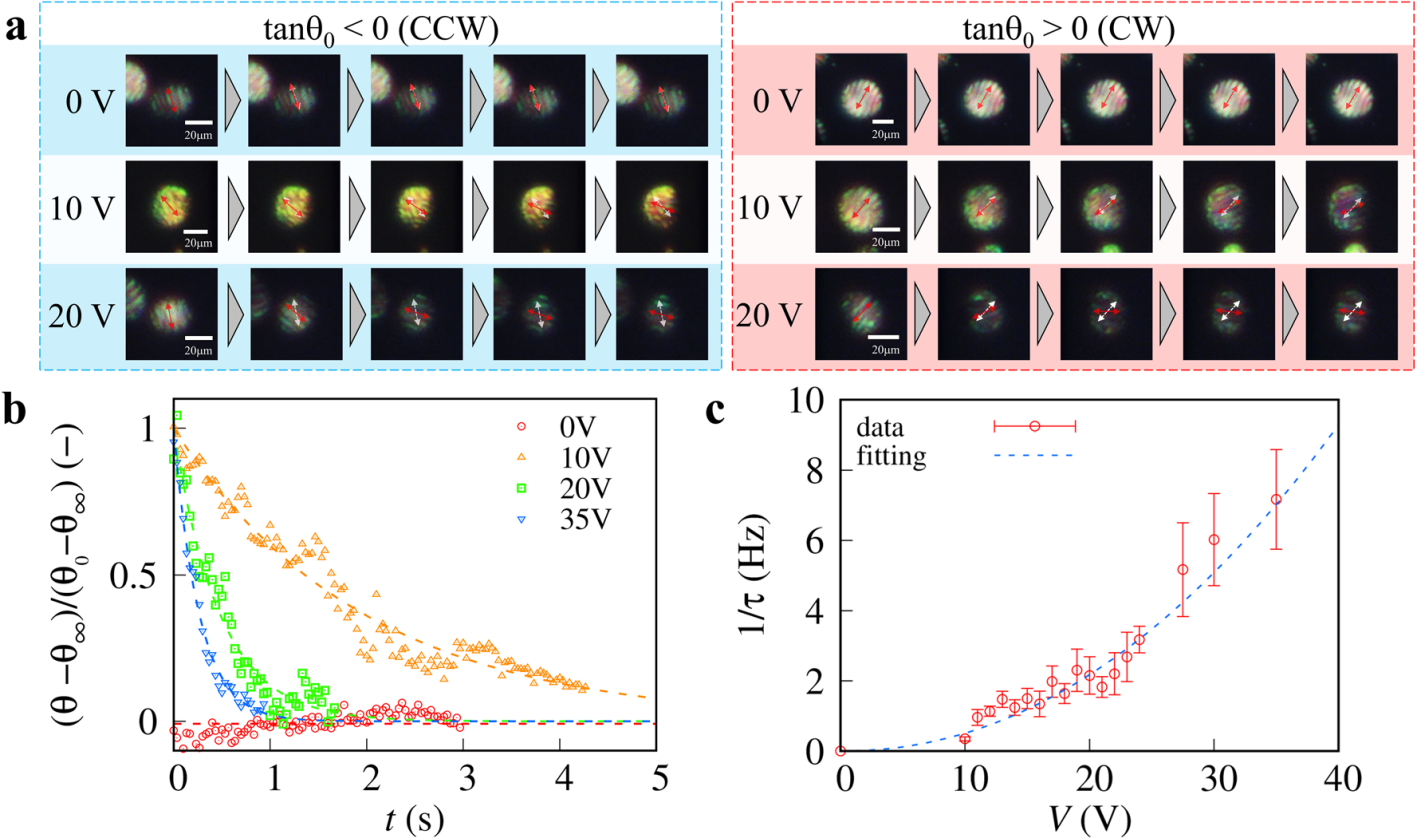
Applied voltage dependence of rotational behavior of LCDs. **(a)** POM images of LCDs under voltages of 0, 10, and 20 V. The sign of tan θ_0_ determines the rotational direction. Images were captured every 0.5 s. We show the direction of ***n*** and the initial direction as red and white dashed arrows, respectively. **(b)** The time evolution of the normalized direction $\overline{\uptheta }$ = (θ − θ_∞_)/(θ_0_ − θ_∞_). The dashed lines represent the best fits for single exponential functions. In the case of 0 V, the direction was normalized as $\overline{\uptheta }$ (0 V) as (θ − θ_∞_)/θ_0_, and the mean is displayed as a dashed line. **(c)** *V* dependence of 1/τ. The dashed line represents the best fit determined by a power function (∞ α*V*^β^). The fitting parameters α and β are 4.1 (± 2.0) mV^−2^ ms^−1^ and 2.1 ± 0.2, respectively.

The relationship between the applied voltage and the rotational speed of LCDs is investigated to establish the distribution of the magnitude of the electric field. POM images of LCDs in response to the applied voltage are shown in Fig. 2a, and to eliminate the position dependence, only the LCDs that are located within the red rectangles in Fig. 1i are visualized. LCDs without voltage (0 V) do not rotate and preserve an initial random θ_0_. However, it has been shown that LCDs rotate in response to voltage application, and that the angular velocity of LCDs operating at 10 V is 0.2 rad s^−1^ irrespective of the rotational direction (CW or CCW). When voltage was increased to 20 V, the angular velocity increased to 0.5 rad s^−1^. The direction of rotation is dependent on θ_0_, and |θ − θ_∞_| decreases monotonically.

To eliminate the impact of the initial angle and the normalized angle of LCDs during voltage application, $\overline{\uptheta }$ = (θ − θ_∞_)/(θ_0_ − θ_∞_). For quantitative analysis of rotational behavior, since voltage-free LCDs do not rotate, θ was normalized with *V* = 0 V as $\overline{\uptheta }$(0 V) = (θ − θ_∞_)/θ_0_. The time evolution of $\overline{\uptheta }$ is shown in Fig. 2b, indicating the decay time of $\overline{\uptheta }$ as the voltage decreases. Thus, the relaxation frequency (1/τ) was estimated by fitting the experimental data with a single exponential function [~ exp(− *t*/τ)]. The experimental results and the fitting result indicate that the rotation of LCDs under voltage relaxes single exponentially. The rotational speed depends on the radius of LCDs *R*. To neglect the effect of the size of LCDs, we analyzed the rotational behavior of LCDs with *R* = 15 µm. Previous works reported that the rotational speed is inversly proportional to size squared^13,16^. Thus, we can renormalize the effect of size as *R*^2^/τ.

The dependence of *V* on 1/τ is displayed in Fig. 2c, showing that the 1/τ increases monotonically as *V* increases. The experimental data were fitted usinga power function of V (~ α*V*^β^, where α and β are fitting parameters) and presented as a dashed line in Fig. 2c. The fitting parameters obtained for α and β are 4.1 (± 2.0) mV^−2^ ms^−1^ and 2.1 ± 0.2, respectively, indicating that 1/t ∞ *V*^2^. In principle, at the location where an LCD is positioned, the size of the electric field should be proportional to the applied voltage (*E* ∞ *V*). The rotating motion equation of LCDs is ν∂θ/∂*t* =  − ∂*F*_el_/∂θ, where ν is a rotational viscosity constant^12,15^. Substituting Eq. (1) into the electrostatic force ∂*F*_el_/∂θ, we obtain ∂*F*_el_/∂θ ∞ Δε*E*^2^. The experimental evidence for $\overline{\uptheta }$ = exp(− *t*/τ) implies that 1/τ should be equal to ε_0_Δε*E*^2^/ν, which is consistent with the dependence of 1/τ (∞ *V*^2^).

The mapping relation between 1/τ and *V* is given as:

2 $${\text{V}} = 0.32 \pm 0.16 ({\text{mV}} {\text{ms}}^{1/2} )\sqrt {\frac{1}{\uptau }}$$

Equation (2) implies that the local voltage of microelectrical devices can be estimated. Substituting typical 1/τ ~ 1–10 Hz and electrode gap *d* = 120 µm into Eq. (2), we obtain the local electrical potential detection accuracy of LCDs as Δ*E* ~ Δ*V*/*d* ~ 4 µV/µm.

### Visualization of electrostatic energy density distribution in microcomb electrodes

**Figure 3:**
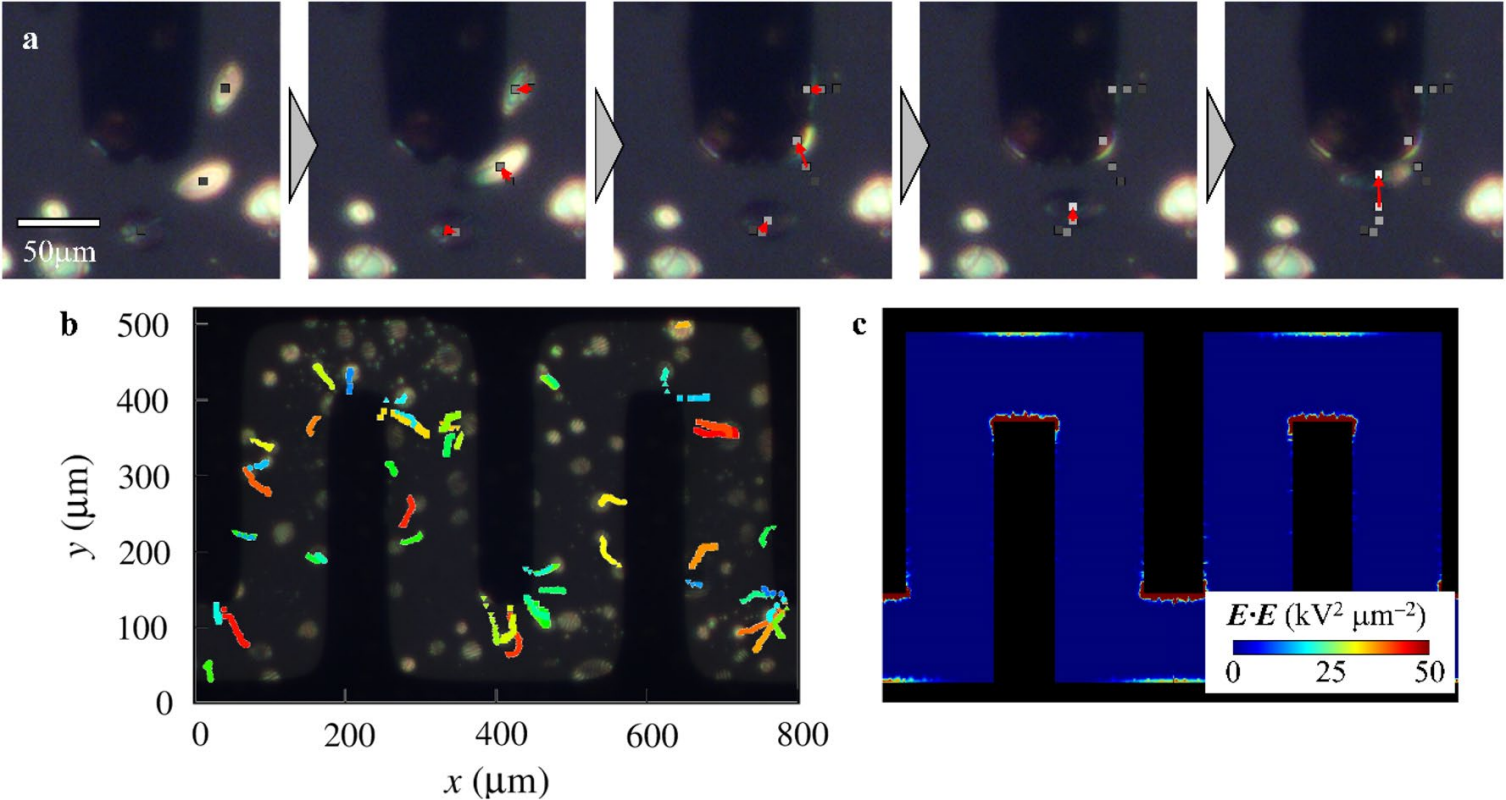
The transportation of LCDs on the microcomb electrodes. **(a)** POM micrographs of LCDs after voltage application. Images were taken every 1/3 s. Square markers and red arrows indicate the centers of gravity of LCDs and deviation from 1/3 s ago, respectively. **(b)** Voltage activated transport of LCDs. We obtained the centers of gravity of LCDs every 0.1 s and superimposed the trajectories on a typical POM image. **(c)** Distribution of electrostatic energy density on the microcomb electrodes from numerical analysis. The color map shows the electrostatic energy density.

When a high voltage is applied to the microcomb electrodes, the LCDs exhibit distinctive dynamics, and the POM images of LCDs are in close proximity to the prominent part of the microcomb electrode obtained with AC voltage of 60 V and 10 kHz as shown in Fig. 3a (Supplementary Movie 2). Using square markers and red arrows, the LCDs’ centers of gravity and the deviation from 1/3 s ago were highlighted. The application of voltage causes the translational motion of LCDs toward the prominent part, and the LCDs are transported to the Au/Cr thin film electrode at equilibrium. Furthermore, as the velocity of translational motion of LCDs increases, the LCDs are closer to the Au/Cr thin film electrode.

The entire region of the microcomb electrodes was examined for LCDs. The dynamics of LCDs with a voltage of 60 V are illustrated in Fig. 3b. Each LCD’s position was recorded every 0.1 s, and trajectories were superimposed on a typical POM image. The most common path for transporting LCDs is through the most prominent regions, followed by the microcomb electrodes’ edges. The LCD has a higher dielectric constant ε_||_ than the iso phase Iso ε_Iso_, and the substitution of transportable LCDs for the Iso phase reduces the electrostatic energy density by ~ (ε_||_− ε_Iso_) ***E***·***E***. Thus, the electrostatic force proportional to (ε_||_ − ε_⟂_)▽(***E***·***E***) drives the transportation of LCDs toward the maximum ***E***·***E***.

The distribution of ***E***·***E*** on the microcomb electrodes was numerically evaluated using COMSOL, and the result is presented in Fig. 3c as a color-coded map. ***E***·***E*** is maximum (~ 50 kV^2^ µm^−2^) at the prominent region, whereas ***E***·***E*** is local maximum (~ 25 kV^2^ µm^−2^) at the edges; these points correspond to the most common destination for LCD transport. Therefore, the LCDs on microelectrical devices are transported along ▽(***E***·***E***), and the equilibrium position of LCDs reflects the maximum value of ***E***·***E***. In addition, LCDs are capable of visualizing the electrostatic energy density and the electric field.

### Periodic modulation of electrostatic energy density distribution for manipulation of LCDs

**Figure 4:**
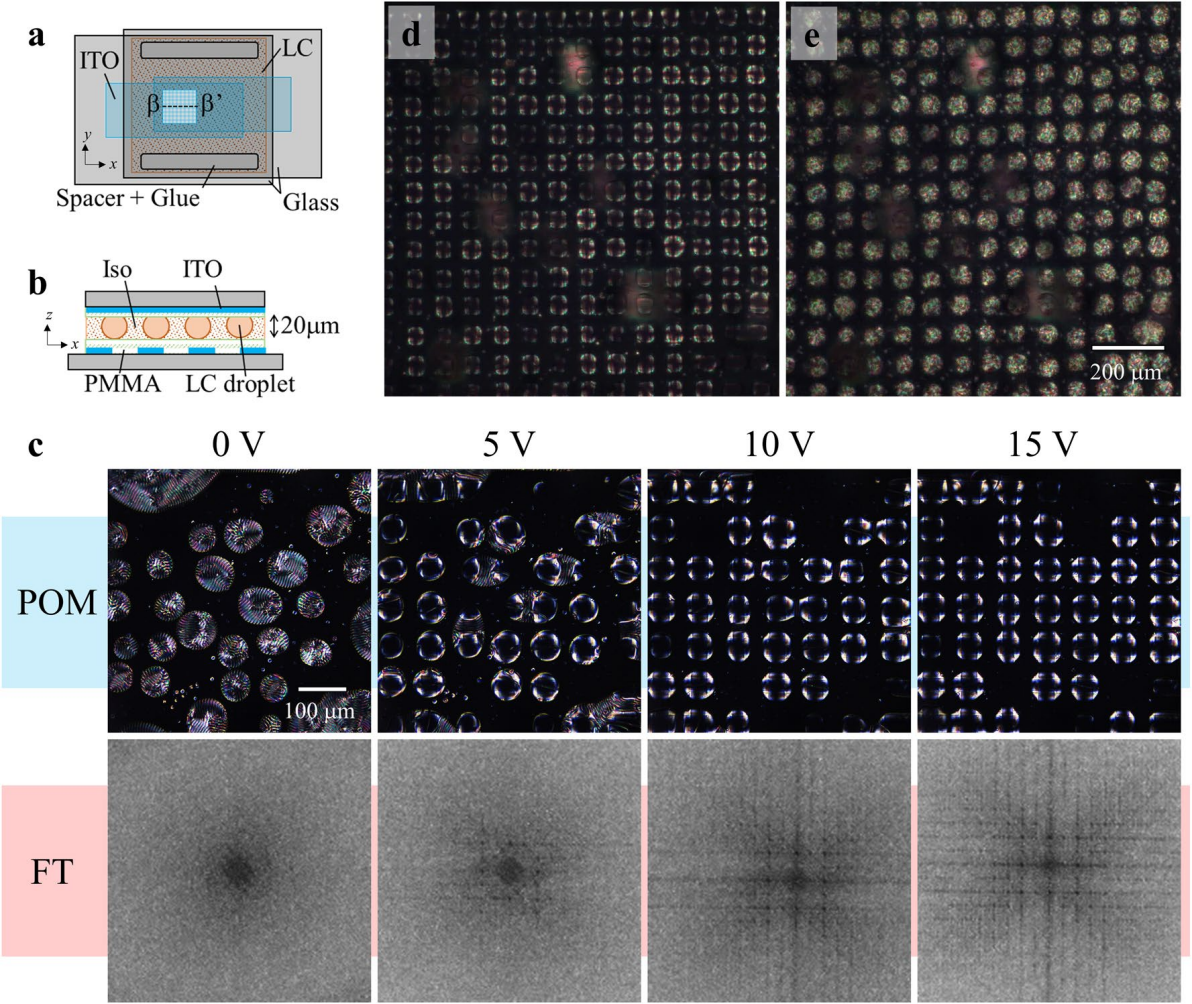
Manipulation of LCDs between micro-ITO electrodes. **(a)** Schematic top view of the microcapacitive device. Designed striped ITO electrodes perpendicularly on upper and lower glass substrates. **(b)** Cross-sectional view (β–β′) of the microcapacitive device. The striped patterns of ITO electrodes on upper and lower glass substrates are parallel to the *y*- and *x*-axes, respectively. We set the cell thickness to 20 µm. **(c)** Morphology of LCDs within the microcapacitive device, POM images with voltages of 0, 5, 10, and 15 V. FT images were obtained by transferring POM images. **(d)** POM image of 15 × 15 LCD array with a voltage of 30 V. **(e)** POM image of LCD array after removing the voltage.

To regulate the position of LCDs through periodic modulation of electrostatic energy density, the microcapacitive device was fabricated and patterned with striped Indium Tin Oxide (ITO) electrodes at interval and a width of 40 µm as shown in Fig. 4a. The LC sample was sandwiched between two ITO-patterned substrates with perpendicular striped patterns of ITO electrodes as displayed in Fig. 4b, whereas Transparent ITO electrodes were used to examine the transmission of the POM. The LC cell was maintained at the Ch-Iso coexisting phase temperature, and the LCDs were dispersed in the microcapacitive device. Figure 4c depicts the POM and Fourier transform (FT) images of voltage-free obtained by transforming POM with ImageJ. From the figure, randomly dispersed LCDs were not observed in the FT image; however, when a voltage of 5 V is applied, a periodic arrangement of LCDs with an interval of 40 µm in the POM image and modest peaks in the FT image are observed. Additional increase in voltage to 15 V leads to higher order peaks in FT images as a result of the order of LCD increasing position.

POM images of LCDs recorded at 30 V and 10 kHz are shown in Fig. 4d, and periodically modulating the electrostatic energy density in the microcapacitive device, a 15 × 15 array arrangement of LCDs was achieved. The ST-director setup is unraveled since LCDs placed in the middle of ITO electrodes are exposed to a strong electric field. After voltage removal, the unique striped texture of Ch-LCs is restored with the layout of array unchanged as in Fig. 4e, indicating that the ST-director setup recovers immediately after the voltage is removed. Here, the nucleation of helixes in each LCD causes defects. To remove defects and recover ST-LCD relaxation or thermal annealing processes are required. These results demonstrate that periodic regulation of the electrostatic energy density facilitates the micrometer-scale arrangement of the refractive index distribution.

**Figure 5:**
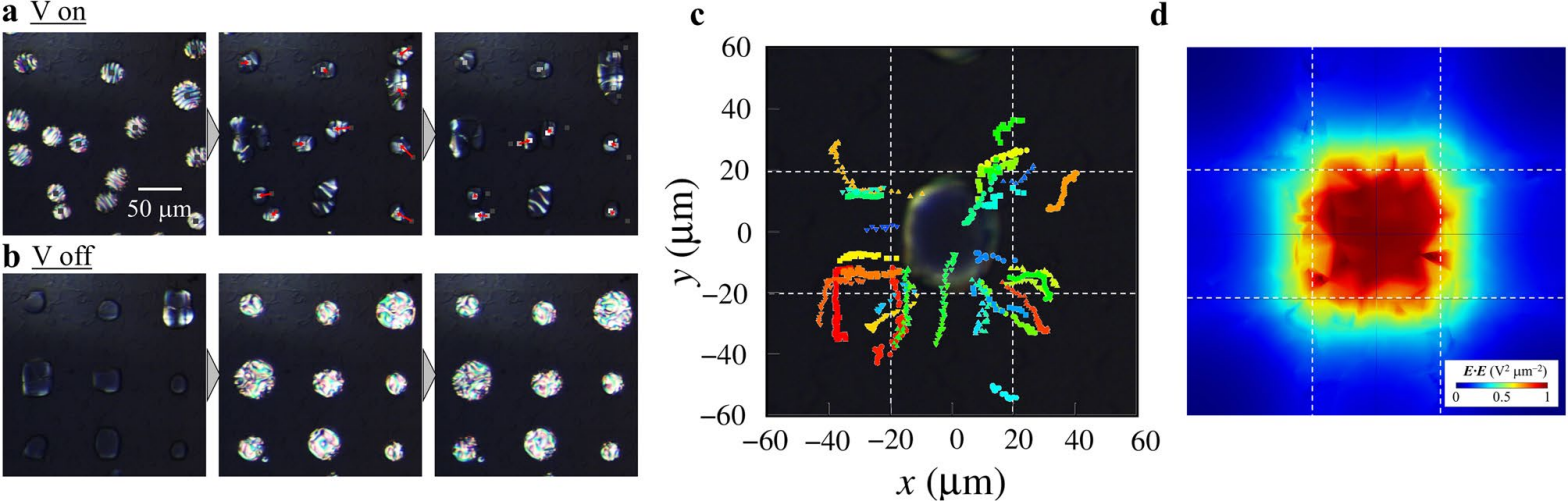
Transportation of LCDs within the microcapacitive device. **(a)** POM images of LCDs after voltage application. Snapshots were taken every 1/3 s. We highlighted the centers of gravity of LCDs and deviation from 1/3 s in square markers and red arrows, respectively. **(b)** POM images of LCDs after removing the voltage. Images were taken every 1 s. **(c)** Trajectories of LCDs in the microcapacitor. We measured the centers of gravity of LCDs every 0.2 s and superimposed the trajectories on a typical POM image. **(d)** The electrostatic energy distribution in the microcapacitive device. White dashed lines represent the ITO electrodes.

The transport dynamics of LCDs between the microcapacitators are evaluated and presented in Fig. 5. Figure 5a illustrates the transportation of LCDs when voltage is applied (Supplementary Movie 3). The LCDs and deviation from 1/3 s are represented by square markers and red arrows, respectively. The LCDs transported in the presence of a voltage exhibited a maximum transport velocity of 60 µm s^−1^, and the ST-director configuration is restored immediately after the voltage is removed as shown in Fig. 5b.

Figure 5c summarizes the repeating transportation trajectories of LCDs. The regions in-between the white dashed lines were patterned with ITO electrodes, and the LCDs are positioned in the direction of the region between the ITO electrodes. We sometimes observed that LCDs are transported into an identical pair of ITO electrodes. In this case, LCDs composed of the same LC materials coalescence with each other. In any case, LCDs are finally located between ITO electrodes. We anticipate that LCDs would be transported toward the location where ***E***·***E*** was maximum in the microcapacitor based on the experimental data of LCD transportation on the microcomb electrodes. Furthermore, the ***E***·***E*** distribution in the microcapacitor was numerically evaluated using COMSOL. The color-coded map of ***E***·***E*** generated by numerical analysis is presented in Fig. 5d. The region between the ITO electrodes is where large ***E***·***E*** occurs, and comparing the experimental and numerical results, we conclude that the electrostatic force ▽(***E***·***E***) accelerates the LCDs in their translational motion and that LCDs move in the direction of the maximum ***E***·***E***. Using a micrometer-scaled periodic modulation of the electrostatic energy density, we successfully arrange the position of LCDs in an array.

### Application of LCD array for heat flux-driven Lehmann rotation

**Figure 6:**
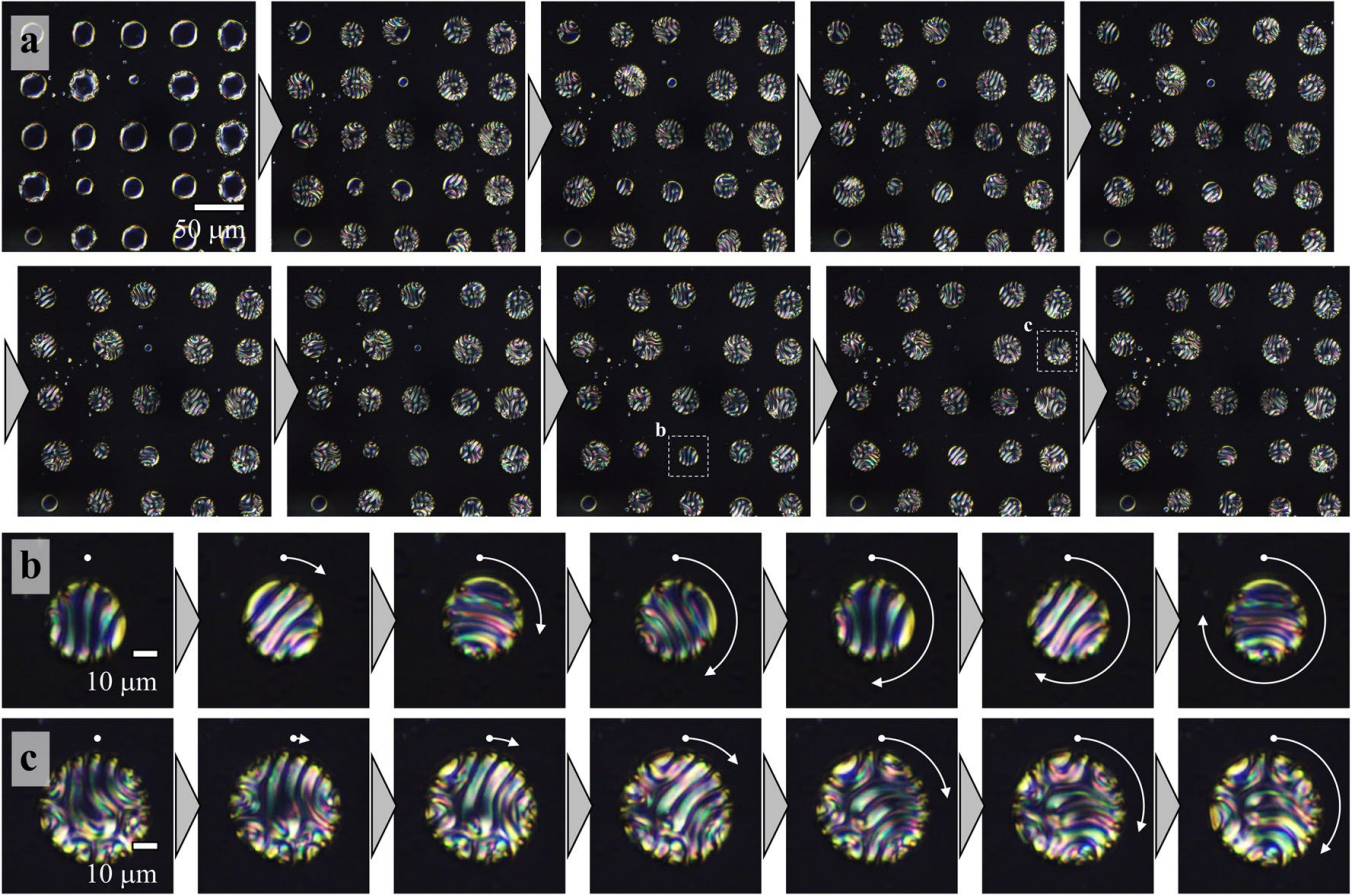
Lehmann rotation of LCD array. **(a)** Rotational behavior of LCD array (5 × 5) with voltage and ▽*T* ~ 5 mK µm^−1^ after decreasing voltage from 30 to 2.5 V. In the micrographs of LCDs surrounded by white dashed lines in **(b)** and **(c)**, white dots and arrows represent the initial position of characteristic texture and rotational angle, respectively. Micrographs were taken every 10 s.

Lehmann rotation is a cross-correlation phenomenon where heat flux through mirror symmetry breaking director configuration is converted into thermomechanical torque^21–25^. In particular, we apply the LCD array technology to this phenomenon and found that in a given heat flux, the LCDs are composed of Ch-LCs and exhibit unidirectional rigid-body rotation^19,26–28^. While providing the microcapacitive device with 30 V, LCDs array configuration was achieved. A temperature difference of 10 K between the top and lower glass substrates was selected and is equal to the temperature gradient (▽*T* ~ 5 mK µm^−1^) and heat flux (*k*▽*T* ~ 0.1 W µm^−1^), where *k* is the thermal conductivity of LCs (~ 0.2 W m^−1^ K^−1^)^29^. To recover the ST-director configuration with the original configuration, the voltage was reduced to 2.5 V. Figure 6a shows the POM images of LCDs subjected to a temperature gradient immediately after decreasing the voltage from 30 to 2.5 V (Supplementary Movie 4). The striped texture was observed after removing voltage, which enabled the ST-director configuration to be recreated. Thus, we extracted two LCDs enclosed by white dashed lines and are displayed in Fig. 6b and 6c. The striped texture of arrayed LCDs rotates unidirectionally in the CW direction, and the relationship between the directions of heat flux and rotation agrees with previous reports; the CW rotation is caused by the upward heat flux through the LCDs with a left-handed twist^13,16,30^. These results suggest that temperature gradient influences LCDs independently and allows the LCD array to rotate in a single direction.

## Discussion

We demonstrate how the rotation and transportation of an LCD can be used to visualize the distribution of electrical properties of microelectrical devices in MEMS, and that the applied voltage to microcomb electrodes causes the striped texture of LCDs to rotate. The equilibrium directions of LCDs agree with the estimated line of electric force from numerical analysis, suggesting that LCDs can detect the direction of the electric field in MEMS. The relaxation frequency of rotation of LCDs was therefore shown to be proportional to the square of applied voltage. The magnitude of the local electric field on the microcomb electrodes with high detection accuracy Δ*E* =  ± 4 µV/µm was determined. Thus, LCDs can be used as PIE sensors to visualize the direction and magnitude of the electric field with a spatial resolution equal to the size of LCDs (~ 10 µm).

Numerical analysis is commonly used to optimize the design of microelectrical devices. Unexpected errors in industrial microelectrical devices often occur during the fabrication process and/or repetitive usage. Since these errors cause the electric field in the fabricated devices to vary from its design, they lead to accidents and malfunctions. Furthermore, it is difficult for numerical analysis to determine the exact location of errors in integrated microstructured electrodes. However, the proposed PIE technique uses LCDs to directly visualize the electric field distribution in the device including errors. The assessment of deviation of the measured electric field from its design facilitates the identification of the errors and increases yield rate and safety.

In addition, we discovered that the translation of LCDs tends toward the maximum ***E***·***E*** value. Comparing the experimental and numerical results reveals that the electrostatic force on LCDs [∞▽(***E***·***E***)], caused by the decrease in the electrostatic energy by replacing the Iso phase with LCDs, is responsible for the transportation of LCDs. Therefore, LCDs can display both the electric field and the electrostatic energy density distribution in microelectrical devices.

Furthermore, we succeeded in arranging LCDs in an array by modulating the electrostatic energy density distribution in microcapacitive devices. This result suggests that LCD transportation can achieve a micrometer-scaled regular array of LCDs with optically unique features different from the Iso phase. To demonstrate the proof of concept, we used the ITO electrodes with a 40-µm interval in this study, and we could arrange an LCD array within a short period. In addition, we proved that electric potential and temperature gradient cause electrostatic force and thermomechanical torque on LCDs and are independent, and when subjected to heat flux, the LCDs rotate unidirectionally perfectly preserving the array conformation. Although a uniform temperature gradient has been used in this study, we can also selectively rotate a portion of the LCD array. Our result shows that the position and direction of a single LCD with a volume of few picoliters can be controlled, and that the array’s configuration can be optimized. A large-scale LCD array with a submicrometer-scaled period could be useful for modulable photonic crystals^31–33^.

## Materials and methods

### Sample and device preparation

Director configuration of nematic LCDs dispersing in the Iso phase is radial because of the anchoring effect at the Ch-Iso interface. Thus, macroscopic dielectric anisotropy of nematic LCD disappears. To recover the anisotropy, we used Ch-LC whose mirror symmetry is breaking. To prepare the Ch-LC sample, 0.93 wt.% of the chiral dopant, (*S*)-4-{[1-(methylheptyl)oxy]carbonyl}phenyl-4(hexyloxy)benzoate, (TCC) was added to the host nematic LC, E7 (LCC). The Ch-LC sample has a left-handed twist director configuration. To expand the Ch-Iso coexisting phase temperature, 9.61 wt.% of octadecane (Sigma-Aldrich) was added to the Ch-LC sample^8,15^. The Ch-Iso coexisting temperature range is 39–41 °C. When we cool the LC sample from the Iso phase temperature to the Ch-Iso coexisting phase temperature, LCDs spontaneously disperse in its Iso phase. In other words, LCDs are composed of the same material as the Iso phase.

Thermal evaporation is used to deposit Au/Cr thin films with thicknesses of 200/50 nm on glass substrates for the fabrication of microcomb electrodes. Photolithography is used to pattern the photoresist, OFPR-800LB (Tokyo Ohka Kogyo Co., Japan) spin-coated on Au/Cr thin film. The electrode width and the interval *d* are 80 and 120 µm, respectively. On microcomb electrodes, we spin-coated poly(methyl methacrylate) (PMMA; Sigma-Aldrich) alignment film. As shown in Fig. 1b, we created LC cells by sandwiching an LC sample between a microcomb electrode substrate and a PMMA-coated glass substrate. The high-speed bipolar amplifier HSA4052 connects both ends of the microcomb electrodes to the arbitrary waveform generator 33210A (Agilent) (NF). We used a sine wave with a higher frequency (10 kHz) than the typical response frequency of LCs (~ 1 kHz), such that the applied voltage can be considered direct current voltage while ionic impurity flow in LCs is suppressed.

To fabricate a microcapacitive device, we spin-coated OFPR on ITO-coated glasses (Sigma-Aldrich) and photolithographed a striped pattern of OFPR with a width and interval of 40 µm. A wet-etching technique is used to pattern the striped ITO electrodes. An LC cell with striped ITO electrodes that are perpendicular to each other after spin-coating PMMA was constructed. The cell thickness is set to 20 µm using glued bead spacers. Since the cell thickness is smaller than the diameter of LCDs, the spatial distribution of LCDs along the *z*-axis is negligible. A high-speed bipolar amplifier was used to connect ITO electrodes and an arbitrary waveform generator, and a 10-kHz sine wave was applied.

### Temperature control and POM

We introduced LC samples into LC cells (microcomb electrodes or microcapacitive devices) using a pipette. In a homemade temperature controller^8^, we used LC cells. Then, we monitored and controlled the temperatures of the upper (*T*_u_) and lower (*T*_b_) substrates, individually. The deviation of temperature from the set value is less than 0.1 K. The thickness of the LC cell is 2 mm. When we set *T*_u_ = *T*_b_, the temperature gradient in LCD cell ▽*T* is guaranteed to be less than 0.1 mK µm^−1^. In contrast, when t T_b_ − *T*_u_ = 10 K, the LC sample is subjected to a uniform temperature gradient ▽*T* ~ 5 mK µm^−1^. We performed transmission POM observation of rotation and translational motion of LCDs using MX9430 (MEJI TECHNO). We used a cross-Nicol polarized at 10° away to distinguish electrodes and the Iso phase.

### Numerical analysis

To estimate distributions of electric field and electrostatic energy density, we used the commercial multiphysics software COMSOL Multiphysics (COMSOL). We selected the unit structure of microcomb electrodes or microcapacitive devices as the calculation area. The experimental high frequency AC voltage is represented by an electric potential difference of ± 10 and the calculated equilibrium voltage distribution. In numerical analysis, we assumed that the difference throughout dielectric constants between LCDs and the Iso phase is small, and that the dielectric constant in the region is uniform. We computed the electric field and electrostatic energy density based on voltage distribution derived from numerical analysis.

## Supplementary Information

Supplementary Video 1.

Supplementary Video 2.

Supplementary Video 3.

Supplementary Video 4.

Supplementary Information 1.

## Data Availability

The data that support the findings of this study are available from the corresponding author upon reasonable request.
