# Supplementary material for: Rotation and transportation of liquid crystal droplets for visualizing electric properties of microstructured electrodes
Source: Sci Rep. 2023 Mar 16;13:4369. doi: 10.1038/s41598-023-31026-8 (PMC10020540; doi:10.1038/s41598-023-31026-8)
Supplement: Supplementary file 5 — Supplementary Information 1. [file 41598_2023_31026_MOESM5_ESM.docx]

Supplementary movies for

**Heat flux sensors using liquid crystal droplets with 0.1-mK-level detection accuracy and high spatial resolution**

Shinji Bono, Satoshi Konishi

*Corresponding author. Email: [bono@fc.ritsumei.ac.jp](mailto:bono@fc.ritsumei.ac.jp)

Supplementary movie 1.

Movie corresponding to Figure 1i. Polarized optical microscopic (POM) movie of liquid crystal droplets (LCDs) dispersion on the microcomb electrodes. We applied an alternative current (AC) of 20 V and a frequency of 10 kHz 3 seconds after the movie started.

**Supplementary movie 2.**

Movie corresponding to Figure 3a. POM movie of LCDs near the prominent part of the microcomb electrode obtained with AC voltage of 60 V and 10 kHz.

**Supplementary movie 3.**

Movie corresponding to Figure 5a. POM movie transport dynamics of LCDs between the microcapacitive device. When we applied a voltage of 5 V, we observed a periodic arrangement of LCDs with an interval of 40 μm.

**Supplementary movie 4.**

Movie corresponding to Figure 6a. POM movie of LCDs subjected to a temperature gradient (∇*T* ~ 5 mK μm^−1^) immediately after decreasing the voltage from 30 to 2.5 V. The recording was performed with 30 times faster speed.
